# A Textbook upon the Pathogenic Bacteria

**Published:** 1904-01

**Authors:** 


A Textbook upon the Pathogenic Bacteria. For Students of Medicine and Physicians. By Joseph McFarland, M. D., Professor of Pathology and Bacteriology in the Medico-Chirurgical College, Philadelphia; Pathologist to the Philadelphia Hospital and to the Medico-Chirurgical Hospital, Philadelphia. Octavo, 629 pages, fully illustrated, a number in colors. Philadelphia, New York, London: W. B. Saunders & Company. 1903. (Cloth, $3.50 net.)

While this work is called a textbook it is more like a story; at the same time it is scientific and serious and is written in such a manner as to be extremely interesting, aside from its value as a reference work on the subject with which it deals. The book is concise in its description of the technical procedures in bacteriology. In nearly every instance a brief account is given of the history of the organism under consideration. Another feature which is most interesting is the description of the pathologic lesions accompanying the invasion of the various organisms. The fact that this is a fourth edition speaks well for the popularity of the book, which has been entirely rewritten and brought up to date. The illustrations, too, are worthy of special mention.

